# Mavacamten Short-Term Hemodynamic, Functional, and Electrocardiographic Outcomes: Initial Real-World Post-Trial Experience

**DOI:** 10.1016/j.jacadv.2023.100710

**Published:** 2023-11-15

**Authors:** Omar M. Abdelfattah, Bradley Lander, Karen Demarco, Karen Richards, Deatrah Dubose, Matthew W. Martinez

**Affiliations:** a Department of Medicine, Morristown Medical Center, Atlantic Health System, Morristown, New Jersey, USA; b Division of Cardiovascular Medicine, University of Texas Medical Branch, Galveston, Texas, USA; c Department of Cardiovascular Medicine, Morristown Medical Center, Atlantic Health System, Morristown, New Jersey, USA; d Director of Sports Cardiology, Department of Cardiovascular Medicine, Morristown Medical Center, Atlantic Health System, Morristown, New Jersey, USA

Despite the increasing recognition that hypertrophic cardiomyopathy (HCM) is the most common inherited cardiomyopathy, the symptom treatment paradigm has remained stagnant for decades. Since the initial description of HCM by Braunwald et al,^1^ our understanding of HCM has evolved from a rare and fatal disease into a contemporary treatable disease. Obstructive HCM (oHCM) phenotype characterized with resting left ventricular outflow tract (LVOT) obstruction constitutes approximately one-third of the patients with an additional one-third HCM patients having a provocable LVOT obstruction.^2^ Beta-blockers and calcium channel blockers are first-line therapy for symptomatic management of oHCM but have not been shown to have a mortality benefit. Patients refractory to medical therapy are eligible for septal reduction therapy (SRT) either with surgical septal myectomy or alcohol septal ablation. Recently, cardiac myosin inhibitors emerged as a new therapy to treat oHCM. Based on the 3 relatively large, randomized trials; phase 3 VALOR-HCM, EXPLORER-HCM and phase 2 MAVERICK-HCM,^3^, ^4^, ^5^ mavacamten was approved by the United States Food and Drug Administration in April 2022 for the treatment of symptomatic oHCM with NYHA functional class II-III symptoms refractory to medical therapy. We present the first real-world single-center experience of mavacamten in oHCM and mid-term clinical, echocardiographic, and electrocardiographic outcomes. This case series followed the reporting guidelines for case series.^6^

This case series included all patients >18 years with symptomatic oHCM treated with mavacamten between May 2022 and February 2023 (n = 23), from a total of 171 newly diagnosed oHCM patients at our institution. Eligible HCM patients for enrollment in our prospective Morristown Mavacamten Registry were >18 years of age, with oHCM; peak LVOT gradient >50 mm Hg either at rest, with Valsalva maneuver, or during exercise; left ventricular ejection fraction (LVEF) ≥55%; and NYHA functional class II with exertional symptoms or class III symptoms. Pregnant patients were excluded from enrollment. Individuals were allowed to continue standard medical therapy. Medications with possible drug-drug interactions with mavacamten were discontinued or substituted with safer alternatives prior to initiating mavacamten. Patients initiated on therapy were those eligible for SRT with refractory symptoms to maximal medical management and opted not to proceed with SRT.

Data on baseline characteristics, echocardiographic, electrocardiographic, and functional characteristics as well, as clinical outcomes were extracted from our prospectively enrolling institutional registry or were manually extracted from electronic medical records. Resting echocardiograms and electrocardiograms (ECGs) were performed at baseline in months 1, 2, 3, 4, 6, and 7 of follow-up. Echocardiographic data included chamber dimensions and markers related to ventricular filling including the ratio between early mitral inflow velocity and lateral (lateral E/e’) and septal mitral annular early diastolic velocity (septal E/e’), average E/e’ ratio, left atrial volume index, and tricuspid regurgitant velocity. Serial LVOT gradient measurements included instantaneous peak LVOT gradient at rest and provoked peak LVOT gradient with the Valsalva maneuver. The ECG parameters included voltage amplitudes in SV1-SV4, RV4, RV5, RV6, and RaVL leads, in addition to T wave inversions and ST-segment changes across precordial leads.

Data are presented as mean ± SD or median (IQR) values for numerical variables and proportions (%) for categorical variables. We used chi-square or Fisher exact tests to compare proportions and Student’s *t* or Mann-Whitney *U* tests for numerical variables. Analyses were performed using GraphPad Prism Version 9.5.1. Any *P* < 0.05, 2-tailed, was considered significant. Box-Whisker plots were used to graphically present absolute changes in diastolic function parameters over time.

In a real-world setting, we present our case series of 23 patients with symptomatic oHCM treated with mavacamten. The mean age was 60.9 years; 14 (60.9%) were women, and 100% were on background β-blocker or calcium-channel blocker monotherapy. Mavacamten was well tolerated with no major adverse events. Four patients experienced minor adverse events (palpitations; n = 2, dyspnea; n = 1, dizziness; n = 1). Six patients (23%) required an increased dose of mavacamten during follow-up to achieve satisfactory reduction in LVOT gradients and symptom management, while 4 patients (17.4%) required a lower dose of mavacamten owing to significant abrupt reduction in LVOT gradients (n = 2), dizziness with postural hypotension (n = 1), and reduction in LVEF from 65% to 55% 4 weeks following initiation of drug (n = 1). Moreover, 8 patients (34.8%) had a dose reduction of their background medical therapy over follow-up. No subjects experienced temporary drug discontinuation, and no patients had a reduction in LVEF ≤50% or were lost to follow-up. Median follow-up was 5.3 months (IQR: 81-207 days). All echocardiograms were reviewed by the senior author (M.W.M.).

**Figure 1:**
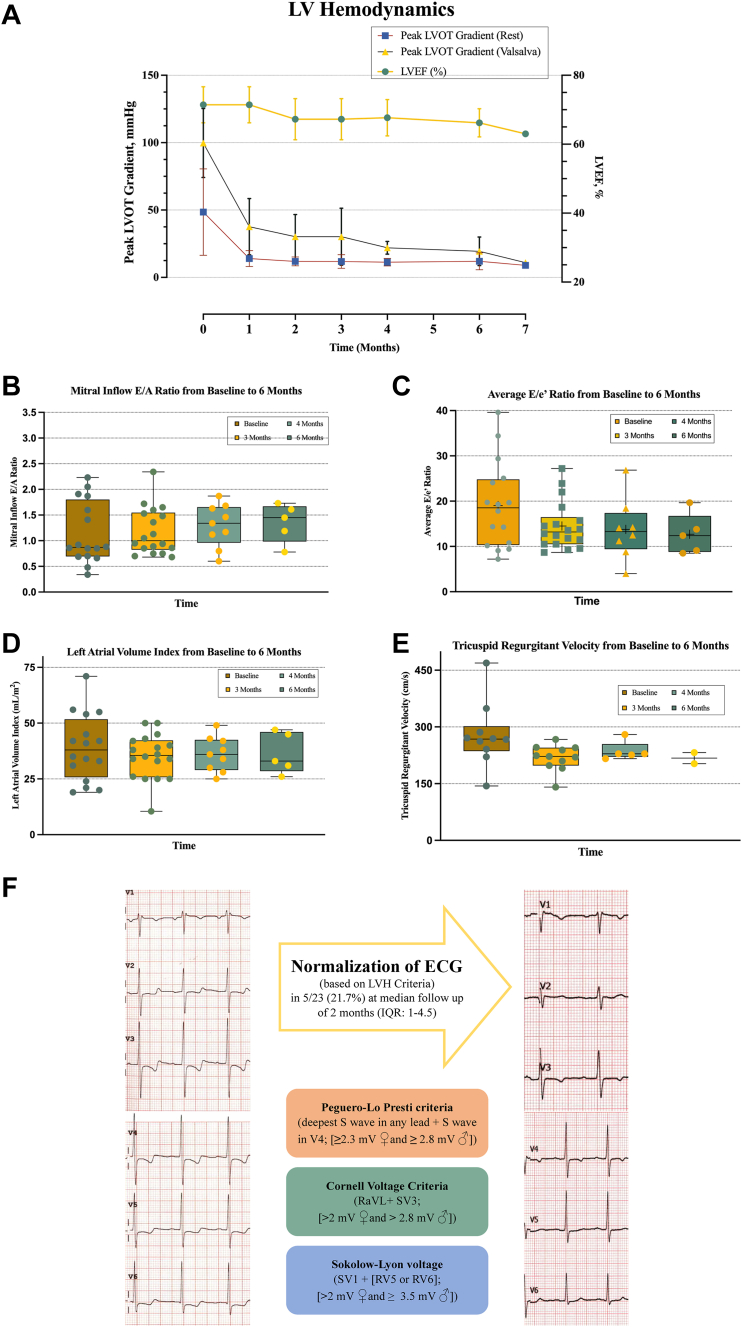
Hemodynamic, Echocardiographic and Electrocardiographic Parameters in Hypertrophic Cardiomyopathy Patients on Mavacamten Throughout Follow-Up (A) Trend of left ventricular outflow tract (LVOT) gradients and left ventricular ejection fraction (LVEF) from baseline to 6-month follow-up. Box-Whisker plots of (B) mitral inflow E/A, (C) average E/e ratio, (D) left atrial volume index, and (E) tricuspid regurgitant velocity at baseline at 3, 4, and 6 months follow-up for patients on mavacamten. (F) Electrocardiographic changes from baseline to follow-up. LV = left ventricular; LVH = left ventricular hypertrophy.

Treatment with mavacamten for 6 months led to a significant reduction in both peak LVOT gradient at rest and with Valsalva. The mean decrease in LVOT gradients at rest from baseline to 3 months was (42.5 mm Hg vs 9.8 mm Hg, mean difference: −32.7 ± 30.5, *P* = 0.008), and at 6 months it was (61 mm Hg vs 9.5 mm Hg, mean difference: −51.5 ± 44.5, *P* = 0.35). The mean decrease in LVOT gradients with the Valsalva maneuver from baseline to 3 months was (98.7 mm Hg vs 32 mm Hg, mean difference −66.69 ± 37, *P* < 0.0001), and at 6 months it was (103 mm Hg vs 19.04 mm Hg, mean difference −83.8 ± 39.9, *P* = 0.009). The mean difference in LVEF from baseline to 3 months was (72% vs 66.4%, mean difference −5.6 ± 7.7, *P* = 0.006), and at 6 months it was (72% vs 66.2%, mean difference −5.8 ± 7.4, *P* = 0.15). The maximal interventricular septal wall thickness was similar between baseline and 6 months (18.17 mm vs 17.94 mm, −0.2 ± 1.8, *P* = 0.60). Patients treated with mavacamten also had an improvement in markers of left ventricular diastolic function (Figure 1). The average E/e’ ratio decreased from baseline to 3 months (19.2 vs 15.5, mean difference −3.7 ± 6, *P* = 0.053), and the difference at 6 months it was (23.9 vs 15.3, mean decrease −8.6 ± 9.9, *P* = 0.27). There was no significant change in the tricuspid regurgitant velocity from baseline to 6 months (241 cm/s vs 222 cm/s, −19 ± 19.8, *P* = 0.40). There was no significant change in left atrial volume index at 3 or 6 months (37.3 mL/m^2^ vs 35 mL/m^2^, −2.3 ± 8.5, *P* = 0.38), and (39.7 mL/m^2^ vs 37 mL/m^2^, −2.7 ± 5, *P* = 0.45), respectively. No significant changes were seen in mitral inflow E and A velocities. Moreover, functional NYHA class improved in 13 (56.5%) patients. Of these 13 patients, 12 (52.2%) had improvement in 1 NYHA functional class, and 1 patient (4.3%) improved in 2 classes.

Serial ECGs with sequential S and R voltage amplitude measurements were obtained. There was no statistical significance difference noted in SV1 voltage amplitude (1.69 mV vs 1.41 mV, −0.2805 ± 0.6, *P* = 0.46), SV2 voltage (2.17 mV vs 1.34 mV, −0.82 ± 0.9, *P* = 0.16), SV3 voltage (2.32 mV vs 1.77 mV, −0.55 ± 1.2, *P* = 0.41), SV4 voltage (1.03 mV vs 1.13 mV, −0.10 ± 0.5, *P* = 0.7), RV5 voltage (2.2 mV vs 1.35 mV, −0.84 ± 0.9, *P* = 0.18), RaVL (0.79 mV vs 0.63 mV, −0.16 ± 0.17, *P* = 0.16). However, RV4 and RV6 voltage amplitude showed a significant decrease (2.33 mV vs 0.77 mV, −1.56 ± 0.87, *P* = 0.03) and (1.26 mV vs 1.05 mV, −0.2 ± 0.01, *P* = 0.04). At follow-up, 5/23 patients (21.7%) experienced normalization of left ventricular hypertrophy (LVH) and strain pattern on electrocardiography based on LVH voltage criteria (Peguero-Lo Presti, Cornell, and Skolow Lyon voltage criteria), with a median time to normalization of 2 months (IQR: 1-4.5 months).

The current case series demonstrated favorable safety and efficacy of mavacamten in a real-world population over a follow-up period of 6 months. This data reflects important clinical experience after landmark clinical trials and is equal to about 20% of the Explorer published data.^4^ The key findings of our study include: 1) excellent mid-term safety profile of mavacamten with a low incidence of minor safety events and no major safety events reported; 2) sustained reduction in peak LVOT gradient at rest and with Valsalva at 6 months; 3) improvement in resting diastolic function parameters; 4) normalization of LVH pattern on ECG in 21% of the population; 5) improvement in NYHA functional class in 56.5% of the population; and 6) minimal reductions in left ventricular systolic function. There was considerable trepidation by the HCM community and the Food and Drug Administration regarding the safety of this new drug class, and frequent echocardiograms have been required to monitor its safety. Given the excellent outcomes and minimal harm demonstrated by this data, it may be reasonable to reconsider the frequency with which echocardiograms are performed in the early period of mavacamten initiation. We suggest an echocardiogram at 6 weeks and 12 weeks while the medication is being titrated upward, and in another 6 weeks if titrated again. Otherwise, we suggest 6 weeks, 12 weeks, 36 weeks, and 1 year for the first year. Additional data is needed to determine if echocardiograms should remain at 6-month intervals or if annual is sufficient. Such a change would decrease the burden this medication has on patients and on health care expenditures.

The main limitation of this study is that it is from a single center of excellence, and the number of patients on this medication in general remains low when compared to other cardiac medications. Second, long-term follow-up was not available at the time of conducting this analysis to assess long-term (>6 months) adverse events and clinical outcomes.

**What is the clinical question being addressed?** What are the effects of mavacamten on the echocardiographic, electrocardiographic, and functional parameters in oHCM?

**What is the main finding?** In a real-world setting, this case series demonstrates that mavacamten improved NYHA classification and LVOT gradients (at rest and Valsalva) while maintaining LVEF. Mavacamten led to the normalization of electrocardiographic parameters for left ventricular hypertrophy in a portion of the population after 4 to 6 months of follow-up. The favorable sustained effect of mavacamten on left ventricular hemodynamics and functional status at mid-term follow-up in this early clinical experience provides insight into the benefits of this medication for oHCM and potentially informs future echocardiographic monitoring frequency.
